# Prize Hospital Report, from the Cheltenham Casualty Hospital. (Prize Adjudged to Mr. Turner.) No. IV

**Published:** 1827-10

**Authors:** 


### PRIZE HOSPITAL REPORT,

### *No.* IV.

MR. CHARLES WILLIAM TURNER, PUPIL.

CHELTENHAM CASUALTY HOSPITAL.

### I.

### CHOREA.

*Treated by Doctor* CRISTIE.

*Case* 1. Ann Turner, æt. 9, was admitted a patient, October 18th. The disease is strongly marked; her arms, legs, and whole body, are continually in motion, partially lost articulation.

She complains of great head-ache, and her bowels are in a very constipated state, tongue white.

R. Hydr. subm. gr. xij. antim. tart. gr. ij. ext. colocynth. c. ℈ij. ℳ et divide in pil. xij. capt. ij. omni nocte.

21*st*. Has taken some turpentine mixture, but her symptoms rapidly increase; she cannot stand without assistance, and is much emaciated; the bowels still rather bound.

R. Hydr. subm. gr. v. pulv. scammon, c. gr. x. ft. pulv. om. noct. sumend. et mane ℥ij. mist. aperien. in usu commun.

28*th*. Medicines have operated very freely, but the involuntary motions not at all lessened. Contin. mist. aper. om. mane, et capt. ℥ss. mist. sequent. bis die. R. argent. nitr. ʒss. aq. menth. p. ℥vijss. syrupi, ℥ss. ℳ ft. mistura.

*Sept. 4th*. Nearly the same as last week, the symptoms not mitigated. Contr. pulv. scammon. c. calomel, et adde ol. terebinth. ℥ss. mistur. c. mucilag. acaciæ q. s. ut antea sumend.

11*th*. The mixture has made her very sick, bowels open, symptoms not at all abated. Capt. ferri carbon. et pulv. jalapæ aa ʒss. bis quotidie.

18*th*. Involuntary motions slightly relieved, augeat ferri carb. ℈ij. et capiat ter quotidie.

25*th*. Symptoms considerably better, can walk a little, abdomen enlarged; bowels rather costive. Rep. pulveres, et capiat ℥ij. mist. aper. in usu commun omni mane.

*Dec. 2nd*. Bowels very regular—walks with firmness. Pergat.

9*th*. Appears in every respect quite well, and latterly to have gained flesh. Dismissed cured.

Out of the great number of cases which have been admitted into this hospital, I never saw one more obstinate. In the beginning of the disease the girl was excessively weak and emaciated, and the remedies which were used very much increased her debility. The symptoms did not at all yield to the turpentine, argent. nitr. or indeed any of the medicines which were prescribed, till she began taking the carbonate of iron, and it was astonishing to see the good effect it had upon her, both with respect to her looks and the irregular motions of her body.

I have seen her since she has been discharged; she has never had any return of her disease, and is now a stout healthy girl.

CHOREA.—*Treated by Doctor* GIBNEY.

*Case* 2. Samuel Clapton, æt. 23, was admitted a patient, January 23rd. Some months ago, (June last) he was riding on a horse, that was drawing a water barrel, when by a sudden start of the horse it threw him off as also the barrel, which passed over his left shoulder across the scapula; there was no external bruise, no deformity, nor was any bone broken; but on rotating the arm a crepitus was sometimes heard over or near the acromion of the scapula, as if a string jerked suddenly off the process.

He has general symptoms of chorea, uses his arm with some difficulty, and his fore-arm with still more. Tongue white, bowels costive, pulse slow; and, although he answers questions, yet he hesitates, and has a very vacant stare; clasps his fingers weakly. Appl. empl. cantharid. nuchæ, et postea ung. sabinæ.

R. Ext. sem. colch. aloes spicat. a ℈iss, pil. hydrarg. ℈ij. pulv. opii, gr. vj. ♏ ft. in pil. xxiv. divid. sum. j. ter quotidie.

*February 6th.* Has to-day considerable twitching of his hands, functions pretty regular. R. Liq. arsenicalis, ♏ lxv. vin. sem. colchici, ʒij. aq. fontis, ℥vj. syr. papav. ʒvj. ♏ capiat ℥j ter quotidie et pulv. jalapæ c. ʒj. 2d quaque nocte.

*16th.* Much the same as last report. Rep. mist. addend. liq. arsenicalis, ♏ xvj. R. Ol. terebinth. ℥j. p. acaciæ, ℥ss. aq. cinnam. ℥v. syrupi, ℥j. ♏ et capiat ℥iss. omni mane.

*27th.* Makes but slight progress. Inf. ung. antim. tartar, nuchæ.

*March 13th.* Is rather better since he used the ointment. Pergat.

*April 3rd.* Has remained as last report, if any change, worse; still some discharge from the ointment; complains of giddiness in the head. Fiat v. s. ad ℥xx. Rep. pil. colch.—omit. mist. arsen.

*April 10th.* Has not made much progress of late; sores dried up; head relieved. Rep. mist. arsenic et terebinth.

*17th.* As usual not at all better. Capiat ferri carb. ʒj. ter in die.

*May 1st.* Has derived much benefit from the iron; in every respect stronger; tongue furred, but bowels regular. Pergat.

*22nd.* Improving. Pergat.

*29th.* Has continued improving; is able to dress himself, and to work a little as a labourer; he is losing the jerking, and uses his hand. Capt. ferri carb. ʒiss. ter quotidie.

*June 5th.* Has still continued to gain strength from the iron; capt. ʒij. ter quotidie.

*July 3rd.* Has gone on well, and is now able to resume all his usual employments; his countenance is altogether more intelligent, and he does not hesitate in his answers as formerly. Dismissed cured.

CHOREA.—*Treated by* DR. CHRISTIE.

*Case* 3. Winifred Justin, æt. 8, was admitted a patient, Feb. 17th. She complained of head-ach; bowels hard, and very much bound; tongue furred; she is constantly affected with involuntary motions of the superior and inferior extremities, but more particularly of the right side; the motions cease during sleep. R. Hydr. subm. et ext. colocynth. c. ā gr. v. in pil. ij. om. nocte sumend. et mane sequent. ℥iss. mist. aper.

*24th.* The medicines have acted very much, but the symptoms are nearly the same as last report. Cap. ferri carbon. et pulv. jalapæ, aa gr. x. bis quotidie.

*March 2d.* Bowels pretty regular; does not appear to be any better from the medicines. R. Ferri carb. ℈j. Pulv. jalapæ, gr. x. ♏. Ft. pulv. ter quotidie sumend.

*10th.* Involuntary motions alleviated; can walk steadier; bowels rather costive.

Rep. pulv. add. pulv. jalap. gr. x. sing. dos.

*17th.* Much better. Pergat.

*24th.* Improving. Bowels regular; tongue furred. Increase the ferri carb. ad ℈ij. and take it, as before, with the pulv. jalapæ.

*21st.* Involuntary motions are now nearly subsided; the powders have kept the bowels freely open, and she can walk with firmness. Pergat.

*April 14th.* She appears perfectly well, and can work with her needle quite steadily. Continue the powders one week, and to be then discharged.

*21st.* Dismissed, cured.

---

### II.

### CASE OF CEPHALALGIA IN WHICH IODINE WAS EXHIBITED.

*Treated by* DR. GIBNEY.

Ann Slater, æt.24, was admitted a patient September 26th.

She complains of severe pain in the head, extending across the forehead, particularly on the right temple, which became worse at night; pupils dilated, even on exposure to light, and she seems of a very heavy disposition; pulse, tongue, catamenia, bowels, all regular; has suffered some months; never gets giddy. Appl. empl. cantharid. nuchæ, et postea ung. sabinæ. Capt. pil. hydrarg. et ext. aloes spic. aa gr. iij. omni nocte et mist. aper. ℥ij. omni mane.

*October 3d.* No improvement. Pergat. R. Ext. belladonnæ, ℈j. Cerat. cetacei, ℥j. ℳ. ft. ung. tempori applicand.

*17th.* The ointment *at first* gave decided relief, but lost its influence; her general health good. Capt. pil. aloes c. gr. x. o. n. et decoct. aloes c. ℥iss. omni mane.

*21st.* The medicines opened her bowels freely, but pain in the head as bad as ever. Appl. hirudines vj. tempori.

R. hydrarg. subm. gr. iijss. Opii, gr. jss. Ft. pil. omni nocte sumend.

*24th.* The calomel and opium pills salivated her, which did her a little good, and the leeches relieved the pain for a time; her eyes have not the fixedness with dilated pupil as formerly; functions continue natural. Rep. pil. et hirud. capiat mis. aperien, ℥iss. omni mane.

*November 15th.* Has (to use her own expression) continued better and worse, except when salivated, which certainly relieved her a little.

R. Tinct. iodinæ, ʒiss. Inf. calumb. ℥vij. Tinct. sennæ, ʒvss. ℳ. Capiat ℥j. ter quotidie.

*December 7th.* The iodine seems to have entirely removed her complaint, and she was discharged cured.

Most probably there was some pressure on the optic nerves, which was removed by the influence of the calomel and opium, or iodine, or, perhaps, both; but she had taken the iodine only four days before she received great benefit.

---

### III.

### GONORRHŒA, WITH IRRITABLE SORES.—*Treated by* C. Averill, *Esq.*

*Case* 1. Patrick Keefe, æt. 21, a labourer, was admitted a patient March 3d.

He states that his complaint first came on with a discharge from the urethra, and a scalding in making water, and that, for some time past, he had not been able to draw the fore-skin back. A fellow-labourer gave him some pills, which contained a great quantity of mercury, and salivated him. Mr. A. to-day divided the prepuce with a phymosis-knife, and found two or three irritable sores on the glans, and under-surface of the prepuce, in a sloughy state. He was ordered to apply lint, dipped in a lotion, composed of a drachm of nitric acid to a pint of water, over that a poultice of beer and oatmeal; allowed to drink a pint of beer daily; and to take ℥j. of the following mixture three times a day. R. Quinin. sulph. gr. xviij. Acid. sulph. dil. ʒij. Decoct. cinchonæ, ℥xij. ℳ. Ft. mistura.

*March 6th.* Appearance not improved. Apply strong nitric acid to the whole surface; continue the poultice, and take the beer and mixture as before.

*14th.* The sores increasing in size; repeat the application of the strong acid, dress it with resin cerate and the same poultice. Pergat.

*17th.* Sloughs caused by the acid separated; sores looking red and healthy, except on the margin of the frænum; the whole surface to be again touched with the acid, and continue as before; he complains of getting no sleep at night, for which, let him take one grain of crude opium in a pill.

*April 10th.* Some inflammation extending towards the body of the penis. Let six leeches be applied, and immediately afterwards a poultice. Sleeps better.

*23d.* From this time, the sores healed rapidly, and in a fortnight he was discharged cured.

This man had taken mercury during the inflammatory stage of gonorrhœa, and had been salivated. Mercury, among the lower orders of Irish, is generally resorted to for the cure of clap; of this class of people there are many in Cheltenham, and cases of the above description are by no means unfrequent. Two or three applications of the strong acid generally arrests the progress of sloughing.

### *Case* 2.—*Gonorrhœa, with Irritable Sores. Treated by* C. Averill, *Esq.*

William Sullivan, æt. 22, was admitted a patient, April 19th. This was precisely a similar case, in every respect, to Keefe's; he had phymosis, discharges from the urethra, and pain in making water. This man had also taken mercury during the inflammatory stage of gonorrhœa, and had been salivated.

*January 21st.* Mr. Averill divided the prepuce, and found three irritable sores in a sloughy state, on the glans: a lotion of nitric acid, in the proportion of twenty drops to six ounces of water, was ordered to be applied, and a poultice of beer and linseed meal—to drink a pint of porter daily. R. Quinin. sulph. gr. viij. acid. sulph. dil. ʒss. Tinct. cinnam. ℥ss. infus. rosæ, vijss. ♏. ft mist. sumat. ℥j. ter quotidie.

25*th*. The sores looking very unhealthy, with thick sloughs: apply the strong nitric acid, dress it with wax cerate, and continue the poultice, &c. R. Opii crudi, gr. vj. et divide in pil. vj. capiat j. omni nocte.

*May 7th*. Sloughs partially come away; repeat the application of the strong acid. Pergat.

14*th*. Sloughs entirely separated, sores less, and looking healthy. Continuent. mistura, &c.

21*st*. Sores still less, and nearly healed. Pergat.

27*th*. Dismissed cured.

---

IV.

CASE OF ANEURISM OF THE AORTA.—*Treated by* HENRY FOWLER, *Esq.*

*September* 14*th*, 1825. JOHN LEECH, æt. 60, a man of choleric temperament, and a school-master, was admitted a patient of Mr. Henry Fowler's. About three months previous to his application for relief, his temper had been roused to more than an ordinary pitch of excitement by the misconduct of one of his pupils, and, according to his own account, after his anger had abated, he became sensible of a sharp and lancinating pain in the interior of his chest; mental excitement or bodily fatigue of any kind after this increased his sufferings to such a degree, as not only to render him apprehensive of danger, but obliging him actually to overlook, and sometimes even to avoid noticing, the negligence and misconduct of his pupils, fearing that, if surprized into a fit of anger, the circumstance might prove highly painful, if not absolutely fatal to him. Till the present period, he had lulled himself with a hope, that a strict attention to a quiescent state of mind and body might eventually relieve him of his symptoms; but this expectation was disappointed by the appearance of a small and pulsating tumour, situated on the right side of the sternum, and projecting in a spot corresponding with the cartilage of the third rib. With this tumour arose two fresh symptoms, one of which he compared to sudden gushes of water in the chest, and the other was, that if, by accident or intention, the swelling was pressed, it immediately induced a violent fit of coughing, and added considerably to the severity of his symptoms. It was at this stage of his complaint that remedies were first administered, but they could only be regarded as palliative, since they merely mitigated his sufferings, without arresting in any degree the progress of the disease: occasional venesection, with the use of the digitalis, were found the most efficient in affording him a degree of comparative comfort and ease, and were, consequently, persevered in to the last. In March, 1827, about a year and a half from the commencement of his complaint, the swelling had increased to the size of a breakfast-saucer, in which strong and forcible pulsations were perceptible: with this increase of size, the symptoms became proportionally augmented; and, on the 14th of the same month, his sufferings were terminated by a rupture of the sac internally.

On dissection after death, which was sanctioned by the request of the patient when alive, the right lung was found adhering to the sac, and forming a considerable proportion of its parietes; the cartilages of the third, fourth, and fifth ribs, with their anterior osseous portions, and part of the sternum, were absorbed, (vide plate) and the fore part of the sac was filled with a great quantity of fibrine, arranged in a succession of concentric layers, and forming altogether an homogeneous and dense mass, of the size of a large orange; this, with the superjacent muscular and cutaneous coverings, were removed from the right side of the sac, and its internal capacious cavity exposed. A wash-hand basin full of coagulated and fluid blood was taken from the chest.

This preparation is preserved in the museum attached to this hospital, and we believe we may add, without fear of imputation for vanity or presumption, that it forms one of the best and clearest specimens of this disease in the kingdom.*

---

* The drawing may be seen at the Editor's.

V.

INJURY OF THE HEAD.

*Treated by* CHARLES FOWLER, *Esq.*

Robert Freeman was admitted into the hospital, in consequence of an injury of the head. The patient was emptying a dry well; he had filled the bucket, and a labourer on the top had nearly drawn it up, when by some accident it slipped, and the whole weight fell with great force on the patient's head; he went on with the work for as much as an hour after the accident, apparently without inconvenience, except a little pain over the eyes. He, however, came down to the hospital, and ℥xvj. of blood were taken from the arm, and a dose of purging medicine given him; he felt the pain in his head quite gone.

*April* 21*st.* Early this morning Dr. M'Cabe was sent for (the man having been previously at work for him.) I went with him, and found him in a very dangerous state, his face flushed, pulse extremely full and irregular, tongue furred, and he could not raise his head from the pillow. I again bled him to forty ounces with great relief. He was immediately afterwards brought to the hospital.

Mr. C. Fowler saw him at 12 o'clock, his face still very much flushed, pulse not so hard and a great deal more regular, pupils dilated, is a little delirious, and complains of great pain in the occiput; there was a slight cut in the scalp on the fore part of the cranium about two inches in length, but no fracture. His bowels have not been opened these three days. Ordered to take five grains of calomel, with a scruple of cathartic extract immediately, and ℥ij. of house mixture every second hour until the bowels have been freely evacuated, a large blister to be put to the nape of the neck, the head to be shaved round the wound, and apply a poultice.

7 *o'Clock* P.M. The medicines have acted very copiously on his bowels; to take six grains of James's powder at 8 o'clock.

22*d.* Delirium gone off, pupils still dilated, he has not such a wild look, but complains of great pain in the back part of the head. Mr. F. opened the temporal artery, and abstracted about ℥x. of blood.

23*d.* He says he felt more relief from the blood taken from the head, than the whole quantity before; pain quite gone; repeat the James's powder to-night.

24*th.* He reports himself to-day quite well, and wishes to get up; tongue still a little furred, bowels open; repeat the James's powder with the addition of 2 gr. of ext. hyosci.

26*th.* Allowed to get up and take a little boiled mutton for dinner; his bowels rather bound; to take calomel and James's powder, of each 4 grains, at bed time, and ℥ij. of house mixture in the morning.

*May* 6*th.* Discharged cured.

---

VI.

INJURY OF A NERVE BY BLEEDING.

*Treated by* C. AVERILL, *Esq.*

Jane Sollis, æt. 38, was admitted a patient, December, 25th. She had been bled in the right arm about a fortnight before; she states the orifice healed very well, but a day or two after her arm felt stiff and uneasy: thinking it rheumatic, and that it would go off, she neglected to apply for relief, until her arm got so excessively painful and heavy, that she could get no rest either night or day. A blister was directed to be applied.

30*th.* The arm more painful; to take one of the following pills night and morning.

R. Hydrarg. Subm. gr. ij.

Pulv. Opii. gr. $\frac{1}{2}$

Cons. q. s. ft. pil.

*January* 2*d.* The arm extremely painful and cannot bear it to be touched; ordered to use the following embrocation frequently.

R. Extract. Belladonnæ, ʒij.

Lin. Saponis. c. ℥ij. ♏ ft. liniment.

9*th.* She thinks she was relieved when applying the liniment, but the pain was soon after quite as bad; equal parts of soap cerate and extract of belladonna, mixed, spread on leather, and bound round the arm in strips.

16*th.* The arm by far less painful, not so tender to the touch, and she can sleep better at night. Pergat.

23*d.* Considerably better in every respect. Pergat.

30*th.* Can straighten her arm without pain, and to-day discharged cured.

### VII.

### CASE OF FRACTURED THIGH ACCOMPANIED WITH HEMOPTYSIS.

*Treated by* CHARLES FOWLER, *Esq.*

Augustus Mayo, æt. 25, was admitted into the hospital, March 16th, for a fractured thigh, occasioned by the fall of a heavy piece of timber. On his admission he was observed to possess all the visible characteristics denoting consumptive diathesis, such as contracted chest, pallid cheeks, with occasional faint hectic flushes, together with that pearly whiteness of the sclerotica which is observed almost invariably to accompany the complaint.

For the first three weeks after his admission, he appeared to be going on extremely well, and then began to complain of a slight pain in his chest, which was shortly afterwards accompanied with an expectoration of mucus tinged with considerable quantities of scarlet blood: as his pulse at this time denoted strong arterial action, he was ordered to be bled, and this seemed to mitigate the pain, though the spitting continued to the same extent as before. A mixture of infusion of roses was now ordered him in combination with the digitalis, and an increased quantity of sulphuric acid, and the tartar emetic ointment to be rubbed upon the chest; after a perseverance in these remedies for some time, the symptoms gradually abated, and, about the sixth or seventh week, his splints were removed in order to ascertain the strength of the osseous union: the callus had been deposited so profusely as to form round the fractured extremites of the bone a globular mass nearly equal in size to the head of a new-born infant, but so deficient was it in the earthy material, that the weight of the leg itself was sufficient to bow the thigh at the fractured part: the thigh was now adjusted as before; the spitting suddenly ceased, the appetite, appearance, and strength rapidly improved; and, at the end of three weeks, the man was enabled to use his crutches—it is now the 12th week, and he can bear his weight to the ground, but the callus is diminished nearly one half its original size.

*Remarks.* This case is curious in as much as Nature had furnished one part of the osseous cement very liberally, even at the time the patient was suffering from hemoptysis; but when this symptom had entirely ceased, the earthy matter was abundantly supplied, and a large portion of the cementing medium removed.

---

### VIII.

### CATARACT.

*Treated by* CHARLES AVERILL, *Esq.*

Mary Bryant, æt. 60, has had cataract in the right eye about three months. The left eye had been operated on for the same disease about a year ago, the pupil of which was closed; directed to take ten grains of Plummer's pill every night.

*Nov.* 18*th*. The operation was performed with Saunder's needle, which was introduced through the sclerotica, about three or four lines from the margin of the cornea; with this the lens was cut into several pieces, one of which was pushed into the anterior chamber. An anodyne draught was given. Extract of belladonna was applied daily, during the continuance of the slight inflammation caused by the operation, and purgatives given occasionally.

*Jan.* 13*th*. A considerable degree of absorption had taken place round the margin of the lens; the light was stronger, and when the belladonna was applied, a space round the margin of the pupil was clear, and the portion of lens which had been left in the anterior chamber was absorbed. To-day the operation was repeated.

*Feb.* 24*th*. A small portion of the hardest part of the lens remaining in the centre of the pupil, the operation was again repeated; this portion was depressed into the vitreous humour; a small part of which, in nearly a fluid state, escaped when the needle was withdrawn. In a few days the inflammation caused by the operation subsided, and she was discharged, having very good vision.

---

### IX.

### AMPUTATION OF THE THIGH THROUGH THE TROCHANTER.

*Treated by* CHARLES AVERILL, *Esq.*

Simon Spicer, æt. 17, admitted on the evening of May 29th. He had suffered some time from an inflammation of the synovial membrane of the knee-joint; at the time of his admission suppuration had taken place to a very great extent; the matter extended along the tendon of the rectus muscle, and on the inside of the thigh nearly to the groin; he was hectic, and had every bad symptom usually attending the formation of large quantities of matter; over the great trochanter the integuments had ulcerated; from the pressure of that part of the bone, all the soft parts being very much wasted; the same was the case on the sacrum. The case altogether was regarded as nearly hopeless; however it was thought amputation of the limb, provided he could survive the operation, might afford him a chance of recovery.

On the 30th, the operation was performed; the artery being compressed in the groin, a narrow-bladed catlin was the knife used, and two flaps, including as much muscle and integuments as the diseased parts would admit, were formed by thrusting the instrument through the soft parts of the thigh on each side of the bone, and cutting from within outwards; the bone was sawed through the trochanter. Very little blood was lost during the operation; four vessels were secured, when he was put to bed quite faint; some wine was given him, and half an hour was suffered to elapse before the flaps were united, which was effected by means of two sutures and adhesive straps. Small doses of opium and ammonia were given at short intervals, and beef-tea and wine were allowed him; towards evening he recovered, was free from pain, and expressed himself as feeling comfortable.

On the 31st, secondary hemorrhage came on to such an extent, as required the stump to be opened; another vessel was secured; he again became faint; musk, wine, ammonia, &c. were occasionally given, which kept him alive to June 1st, eleven o'clock, P. M. when he died.

Had not the secondary hemorrhage come on in this case, it was thought he would have had a fair chance of recovery. On examination of the parts after death, it was found that the small remaining portion of the thigh-bone was healthy. The cartilages, and part of the ligaments of the knee-joint, were destroyed by ulceration.

### *Amputation of the Leg.*

C. AVERILL, *Esq.*

Thomas Guteridge, æt. 37, was admitted into the Hospital June 25th. He had suffered at different times from a small sore on the inner side of the left ancle-joint for the last two years; during which time his general health had been very indifferent, for which he had been treated by Dr. Hawkins, of the Middlesex Hospital. At the time of his admittance into this Hospital there was an abscess as large as an egg, situated on the dorsum and inner side of the foot, which had been opened, and continued to furnish a great discharge of matter; from this abscess sinuses passed in different directions, one leading beneath the muscles of the sole of the foot to the under part of the metatarsal bones; the introduction of the probe gave him such excruciating pain that he would not submit to its being passed either to the ancle-joint, or to those of the tarsus; but, from the external appearance, there was little doubt the bones were diseased. His health was rapidly declining; he was hectic, had profuse night sweats, quick pulse, and irritable cough. Amputation was recommended. In a day or two he made up his mind to submit to the operation; in this time the leg had become œdematous to the calf. The operation was performed rather higher than the usual place below the knee; four ligatures were applied, and he was directed to take ℥j. of the following mixture every two hours:

R. Ammoniæ carbonat. gr. xxiv. liq. ammon. acet. ℥iij. opii sedativ. ♏. xxiv. mist. camphoræ, ℥iij. ♏. ft. mistura.

In a few days the hectic symptoms subsided; he was ordered to omit the mixture as above, and take the one now prescribed; allowed meat diet and a pint of porter daily:

R. Quinin. sulphatis, gr. xv. acid. sulph. dil. ʒss. sacch. albi, ℥ss. infus. rosæ, ℥vj. ♏. et capiat, ℥j. bis die.

All the ligatures came away in less than three weeks.

*Aug. 4th.* The stump is healed, and he waits for his wooden-leg to be discharged.

On dissection of the limb, a sinus was opened leading from the abscess to the ancle-joint; the cartilage of the tibia, as well as the astragalus, was in a state of ulceration, and there was a considerable quantity of pus in the joint.

In cases of amputation below the knee, Mr. A. prefers doing the operation rather higher than the place usually recommended, sawing through the bones immediately below the head of the fibula; by this means the small remaining portion of the leg which projects backwards may be hidden by loose trowsers, and the deformity rendered less than it would otherwise be.

---

### X.

**OSTEO-SARCOMA OF THREE FINGERS.**

*Treated by* Charles Averill, *Esq.*

The patient, Moses Gay, was admitted into the Hospital July 4th. He gave the following account of the disease:

The tumours began forming when he was about four years old, and continued gradually to increase. The skin on the top of the enlargement on the fore-finger, had been some time ulcerated, showing part of the bony tumour in a state of necrosis, and from which there continued to issue a very offensive discharge. There had been, too, a considerable ulcer on the middle finger for some time past. He was occasionally in the habit of correcting the offensive nature of the discharge by the application of nitric acid wash, which had also excited the exfoliation of small pieces of bone. The disease had, to a certain degree, impaired his general health, in consequence of which he had been several times recommended to have the whole hand amputated, to which he would not consent, as, it being his right, he would have been completely disabled from following his trade, that of a carver and stone-cutter: but, when he was told that his thumb and little finger might be saved, he readily consented.

*July 8th.* The three fingers, with part of their metacarpal bones, were amputated by Mr. Averill; who, with a straight-bladed bistoury, dissected back a flap of skin from the dorsum of the hand, then carrying the knife between the metacarpal bones of the thumb and fore-finger, separated them from each other, avoiding as much as possible the muscles of the thumb; the soft parts in the palm being divided, the metacarpal bones of the ring and little finger were separated from each other, and the metacarpal bones supporting the disease sawed through with a metacarpal saw.

It may be worthy of remark, that not a single blood-vessel required a ligature after the operation, though the palmar arches must have been cut. Two or three sutures were applied, and the wound lightly dressed. In about three days considerable hemorrhage occurred, which was stopped by pressure. An abscess afterwards formed in the sheath of the flexor tendon of the little finger, which was opened early, and soon healed.

*Aug. 10th.* He is able to take up and hold small bodies, such as a pencil, &c. and was to-day discharged.

The weight of the three fingers when removed was two pounds. The cavities and interstices within the bony tumours were in the recent state filled with a substance resembling jelly, in which portions of bony matter were deposited. The diseased bony structure forms a beautiful dry preparation, which is preserved in the Museum attached to the Hospital.

---

### XI.

**STRANGULATED FEMORAL HERNIA.**

*Treated by* Charles Fowler, *Esq.*

William Werrett, æt. 60, was admitted into the Hospital May 21st, for a strangulated femoral hernia on the left side.

The operation was undertaken with very little chance of success, as the strangulation (owing to the negligence of the overseers and relations) had existed five days previous to the man's admission; added to this, his appearance was so decidedly cachectic and emaciated, and indicated so low a state of the powers of life, that even if the patient had presented himself at an earlier period of his complaint, the surgeon would have drawn but an unfavourable prognostic as to the final result of the operation.

Too much time had been previously lost, to warrant any further waste of it in the protracted trials of the ordinary means of reduction. The taxis and warm-bath were of course resorted to; but these being found inefficient, the man was immediately placed upon the table, and the operation commenced; the incisions were made after the usual method, viz. a transverse and longitudinal cut through the integuments, and over the tumour, in such a manner as to give the operator the best command of the sac and its coverings; these latter were successively and carefully dissected through, and the sac opened, when a nodule of intestine, and portion of omentum, strongly and extensively adhering to it, were brought to view; a director was then slid under the stricture, and a probe-pointed bistoury passed along its groove up to the strictured part, which was immediately divided by gently urging the latter instrument, in a slanting direction, towards the umbilicus.

Mr. C. Fowler having finished the operation, and the wound being dressed, the patient was carried to his bed; he expressed himself as greatly relieved; his pulse, from an intermitting state, became firm and regular; there was no anxiety of countenance, no swelling, nor tension of the abdomen; and, after repeated doses of purgative medicines, with enemas, his bowels were copiously relieved; indeed every symptom till the next day furnished us some hopes of success. When Mr. F. came the following morning, he found him sleeping, with his mouth open, which, added to his general cadaverous looks, gave him the appearance of a man in the last act of expiration: we closed his jaw, but, on removing the support, it fell again, so completely had the temporal and masseter muscles lost their power of contractility; we looked upon this symptom as pretty strong evidence of the weakened state of the vital powers: on examining the abdomen, it had become quite tense and tympanitic; the pulse was fluttering, quick, and imperceptible; we awoke him from his stupor, but he quickly relapsed into it again: the symptoms of dissolution speedily increased, and, on the following night, the man expired.

*Dissection.* The appearances, on dissection, were not by any means of a formidable character, though the strangulation had existed five days previous to the performance of the operation; there was not the least approach to gangrene in the strictured part; a general and very intense redness, indicating inflammation, was all that could be observed.

Thus, the man seems to have died for want of that energy in the vital powers which would probably have supported a younger and more favourable subject through a severer and more powerful shock.

---

### XII.

### NEW METHOD OF CLOSING THE MOUTHS OF JARS CONTAINING WET ANATOMICAL PREPARATIONS.

The advantage of the method about to be described, consists in its occupying much less time than the one usually employed of tying over the mouths of jars with macerated bladder, &c. it has also a neater appearance; it is adopted by Mr. Averill, who has permitted me to make use of it in my reports from this hospital. The wet preparations in our museum are nearly all preserved in this manner, and many of them have been put up near two years, without any loss of the spirit contained in the jars having taken place. In a pamphlet, the principal part of which is translated from the French, by Doctor Chichester, entitled "Instructions for Collecting, Preserving, and Transporting such Specimens of Natural History as appertain to the Animal Kingdom," the doctor describes a cement used by Monsieur Person, for the purpose of coating externally the corks with which he had closed the mouths of his glass jars. This cement or bite is easily prepared, dries, and acquires complete solidity as soon as applied, it is not acted on by the spirit, adheres to the glass, and does not fall off. It is composed of the following materials.

Pitch, (brai sec des marins)
Yellow wax,
Oil of turpentine, of each two ounces;
Red ochre, well powdered, six ounces.

Let the pitch and yellow wax be melted together, then add the turpentine and red ochre, boil it for a few minutes, taking care that it does not catch fire.

The manner in which Mr. A. uses this cement is as follows. The preparation being suspended in the jar in the situation in which it is intended to remain, a piece of sheet lead (such as is used for lining tea chests) is cut rather larger than the mouth of the vessel, so that it shall overlap the margin or rim round. The cement being melted, the under surface of the lead is completely coated with it by a painting brush, when it is instantly laid over the mouth of the jar, and the border of the lead pressed up smoothly with a spatula over the margin. The cement dries almost instantly, and completely seals the vessel. Mr. A. then paints the surface of the lead with a mixture of lamp-black and black japan, and afterwards with one coat of japan alone.

The accompanying preparation of tubercle of the liver is preserved in the above manner; it may remain with Doctor Johnson for some time, for the inspection of those gentlemen who may feel disposed to adopt the above method: the spirit in this case is rather tinged with bile, and is slightly turbid, owing to the preparation having been put up rather too soon. CHARLES W. TURNER.

*Cheltenham Casualty Hospital, August, 15th, 1827.*

---

